# Circulating cancer-associated macrophage-like cells and macrophage-related cytokines in obese patients with advanced breast cancer who undergo neoadjuvant chemotherapy

**DOI:** 10.7150/jca.89453

**Published:** 2024-09-16

**Authors:** Toshiaki Iwase, Aaroh Parikh, Dong Wenli, Yu Shen, Daniel L. Adams, Cha-mei Tang, Evan N. Cohen, James M. Reuben, Tushaar Vishal Shrimanker, Sudpreeda Chainitikun, Kumiko Kida, Akshara Singareeka Raghavendra, Maryanne E. Sapon, Onur Sahin, Anjali James, Nithya Sridhar, Ann H. Klopp, Debasish Tripathy, Naoto T. Ueno

**Affiliations:** 1 Section of Translational Breast Cancer Research, Department of Breast Medical Oncology, The University of Texas MD Anderson Cancer Center, 1515 Holcombe Boulevard, Houston, TX 77030, USA.; 2 Diagnostic Radiology, Baylor College of Medicine, 1 Moursund St, Houston, TX 77030, USA.; 3 Department of Biostatistics, The University of Texas MD Anderson Cancer Center, 1515 Holcombe Boulevard, Houston, TX 77030, USA.; 4 Creatv Microtech, 9900 Belward Campus Drive, #330, Rockville, MD 20850, USA.; 5 Department of Hematopathology, The University of Texas MD Anderson Cancer Center, 1515 Holcombe Boulevard, Houston, TX 77030, USA.; 6 Department of Internal Medicine, Greenwich Hospital, 5 Perryridge Rd Greenwich, CT 06830, USA.; 7 Department of Medical Oncology, MedPark Hospital, 3333 Rama IV Rd, Khlong Toei, Bangkok 10110, Thailand.; 8 Breast Center, St. Luke's International Hospital, 9-1 Akashi-cho, Chuo-ku, Tokyo 104-8560, Japan.; 9 Department of Radiation Oncology, The University of Texas MD Anderson Cancer Center, 1515 Holcombe Boulevard, Houston, TX 77030, USA.; 10 Department of Breast Medical Oncology, The University of Texas MD Anderson Cancer Center, 1515 Holcombe Boulevard, Houston, TX 77030, USA.; 11 University of Hawai'i Cancer Center, 701 Ilalo Street Honolulu, HI 96813, USA.

**Keywords:** breast neoplasm, macrophages, obesity, neoadjuvant therapy, inflammation

## Abstract

**Purpose:** Cancer-associated macrophage-like cells (CAMLs) are rare, gigantic, and atypical circulating cells found exclusively in the peripheral blood of patients with solid cancers. Obesity-induced hypoxia attracts macrophages to the tumor microenvironment, where they contribute to establishing chronic inflammation, leading to cancer progression. We hypothesized that obese patients with advanced breast cancer may have CAML profiles different from those of nonobese patients, and these profiles may correlate with proinflammatory markers or other macrophage-related markers.

**Methods:** We prospectively collected 20 mL of peripheral blood from patients diagnosed with stage 2-4 breast cancer. We identified CAMLs using the CellSieve microfiltration system and in parallel quantified the proinflammatory and macrophage-related markers using a multiplex cytokine panel. We further evaluated C-X-C chemokine receptor type 4 (CXCR4) expression in CAMLs to investigate its relationship to the macrophage differentiation. We estimated the association between CAML characteristics and body mass index (BMI), body composition, and cytokines/chemokines.

**Results:** Thirty patients were included in the study, and 28 samples were analyzed. Higher BMI was significantly correlated with the increased maximum CAML size (*P* = 0.035). Patients with higher BMIs had significantly increased macrophage-colony stimulating factor (M-CSF) levels in plasma (*P* = 0.007), and obese patients trended towards higher tumor necrosis factor-alpha, MIP-1α and M-CSF expression (*P* <0.10). Body composition analysis showed that the M-CSF and SAT amounts were significantly correlated (*P* = 0.010). MIP-1α expression was significantly correlated with average CXCR4 CAML expression (*P* = 0.003).

**Conclusion:** We discovered larger CAML size was associated with SAT-dominant obesity with increased macrophage-related and proinflammatory markers in obese than in nonobese breast cancer patients.

## Introduction

Approximately 38% of adults in the United States is obesity, which defines as a body mass index (BMI) of 30 kg/m^2^ or more based on the World Health Organization BMI classification 1. Notably, it is significantly more common in women than in men (40% vs. 35%) 1. It is a common, preventable risk factor for metabolic diseases such as hyperlipidemia and diabetes mellites, and it is also significantly associated with increased morbidity and mortality in patients with breast cancer 2, 3. A meta-analysis that evaluated the morbidity risk of obesity in postmenopausal women showed that those with an increase of 5 kg/m² in BMI had a 1.12 increased risk ratio (95% CI, 1.08-1.16) for acquiring breast cancer 4. Obesity also negatively affects breast cancer treatment which has been observed in studies of efficacy endpoints, such as studies of patients with breast cancer who completed treatment with neoadjuvant chemotherapy showing significantly lower pathological complete response rates and shorter disease-free survival durations in patients with BMIs over 30 kg/m² compared to those with lower BMIs 5-7. Further, adjuvant chemotherapy and endocrine therapy studies of patients with breast cancer also showed similar trends in poorer survival outcomes 8.

A potential reason for worse clinical outcomes in patients with higher BMI is obesity-induced chronic inflammation. Adipose tissue has a unique local microenvironment composed of various types of cells, including adipocytes, adipose stromal cells, and immune cells. In the cellular environment of the obese individual, oversized adipocytes induce local hypoxia because of their high oxygen demand, and hypoxia stimulates adipose stromal cells to secrete multiple types of growth factors and pro-inflammatory cytokines ‒ including insulin-like growth factor, vascular endothelial growth factor, interleukin (IL)-8, and IL-10 ‒ which can accelerate breast cancer progression 9. Furthermore, the proinflammatory microenvironment attracts a variety of immune cells, including T cells, B cells, and macrophages, which help establish chronic inflammation.

Although the direct pathological investigation of harvested tissue would be the optimal way to evaluate the inflammatory and immune status of the tumor microenvironment (TME), tissue biopsy is an invasive procedure in most cases. Instead of an invasive tumor biopsy, recent development of liquid biopsy techniques has become a more favorable way to indirectly assess the biology of local tumor. Among the circulating components in the peripheral blood, researchers have investigated cancer-associated macrophage-like cells (CAMLs). These circulating cells are unique, giant, and pleomorphic cells that resemble macrophages for their specificity to cancer, predictive values and prognostic values as biomarkers 10. CAMLs are characterized by specific cell-surface markers such as cluster of differentiation (CD)14 and CD45, cytokeratin, and epithelial cell adhesion molecule; their size (25-300μm); and their atypical, separated polymorphic nuclei (14-64μm in diameter) 10. They are frequently observed, with high sensitivity and specificity, in the peripheral blood of patients with advanced solid cancers 11. Indeed, their significant prognostic value was demonstrated in a study that showed that, in patients with solid tumors, those with more than 6 CAMLs or CAMLs over 50μm in size at initial diagnosis had significantly shorter overall survival and progression-free survival durations than did patients with fewer or smaller CAMLs 12, 13.

Although these previous studies have indicated the usefulness of CAMLs as predictive and prognostic biomarkers, the biological role of CAMLs is not fully understood. Given that CAMLs originate from tumor-associated macrophages (TAMs) 10, and TAMs play an important role in promoting obesity-induced chronic inflammation and tumor progression in the TME, we undertook this study to clarify the biological role of CAMLs and gain a better understanding of how obesity promotes breast cancer progression through systemic inflammation and macrophage-related cytokines. Moreover, we sought to elucidate the mechanism of obesity-induced breast cancer progression, which could aid the development of novel treatments targeting inflammation. Our hypothesis of the present study was that obese patients with advanced breast cancer have CAML profiles that are distinct those of non-obese patients in terms of CAML numbers and sizes. Further, we hypothesized that CAML profiles may correlate with macrophage-related peripheral blood markers and proinflammatory markers. To test this hypothesis, we conducted a prospective study in patients with advanced breast cancer who had undergone neoadjuvant chemotherapy.

## Material and methods

### Materials

This prospective study examined peripheral blood from patients with primary untreated breast cancer taken prior to receiving neoadjuvant or induction chemotherapy (NAC). We prospectively screened the patients in the Breast Medical Oncology clinic at The University of Texas MD Anderson Cancer Center main campus between August 2018 and January 2020. To be included in the study, patients had to be over 18 years old; be newly diagnosed with pathologically confirmed, invasive breast cancer of any subtype (including inflammatory breast cancer); have stage II to stage IV disease; be scheduled to undergo computed tomography (CT) or positron emission tomography-CT before starting NAC; and either not have started chemotherapy or have started it 4 weeks or less prior to enrollment in the study. Both men and women were eligible for inclusion. Thirty patients registered for the study, but 2 samples were of insufficient quality because of clotting or inadequate volumes. Thus, the assay was performed on 28 samples. Patient demographics, pathologic information, and response to the treatment were extracted from MD Anderson Cancer Center's electronic health records system. MD Anderson's Institutional Review Board approved this study (protocol number PA17-0542), and all patients provided written informed consent.

### Detection of CAMLs and cytokines in peripheral blood

We prospectively collected 20 mL of anonymized fresh peripheral blood (two10-mL samples) from each eligible patient after enrollment and stored the samples in ethylenediaminetetraacetic acid (EDTA) or CellSave (MENARINI silicon biosystems, Pennsylvania United States) tubes for the cytokine assay or CAML detection, respectively.

The plasma from blood collected in the EDTA tubes was stored in 2 Eppendorf tubes at -80 ℃ before cytokine/chemokine levels were determined in a single batch using Bio-Plex Pro Human Cytokine 48-Plex Screening Panel (Bio-Rad Laboratories). The cytokine assay focused on proinflammatory cytokines (interleukin (IL)-1b, IL-6, and necrosis factor-alpha [TNF-α]); chemokines: macrophage-related cytokines (macrophage inflammatory protein [MIP]-1 alpha [α], MIP-1 beta [β], and macrophage-colony stimulating factor [M-CSF]).

Peripheral blood was collected in CellSave tubes and passed through the CellSieve™ microfilters that are lithographically fabricated membranes with high porosity, precise pore dimensions, and regular pore distribution to enrich for peripheral blood cells larger than 7 µm 11.

The enriched peripheral blood cells on the microfilter were subjected to staining with 4′,6-diamidino-2-phenylindole (DAPI), anti-CD14 conjugated with Cy5, anti-CD45 conjugated with Cy5, anti-C-X-C chemokine receptor type 4 (CXCR4) conjugated with Alexafluor555, and anti-Cytokeratin conjugated with AlexaFluor488. CAMLs from breast patients were defined as previously described by Adams *et al.* 2014 and Raghavakaimal/Adams BCR 2022), as enlarged (≥30µm in diameter), polynuclear cells with diffuse cytoplasmic cytokeratin and either CD14 positive or CD45 positive. CAMLs were differentiated from CTCs and CD45/CD14 white blood cells (WBCs) as previously described 14, 15. Specifically, CTCs are filamented Cytokeratin positive and CD45/CD14 negative cells. WBCs are cytokeratin negative and CD45/CD14 positive cells. Expression of CXCR4, which is a receptor of C-X-C motif chemokine ligand 12 (CXCL12), was also measured to evaluate the CAMLs's biology related to macrophage-like function because the activation of CXCR4/CXCL12 signaling causes differentiation of monocytes into proangiogenic, immunosuppressive macrophages in the TME 16. CAMLs' size, number, unique morphological characteristics (e.g., merged nuclei), and CXCR4 expression were also evaluated. CAMLs were imaged using and Olympus BX61WI fluorescent microscope with Zeiss AxioCam camera. Images were processed with expression and cell sizes measured using a pre-calibrated sizing tool function in the Zen2011 Blue software (Informer Technologies, Inc. Los Angeles, CA).

### Body composition measurements

To investigate the detailed effects of obesity on the treatment response, we determined patients' body mass composition by characterizing patients' abdominal fat tissue as visceral adipose tissue (VAT) or subcutaneous adipose tissue (SAT). Estimates of the VAT and SAT (both measured in cm^3^) were obtained from CT imaging data provided by MD Anderson's Digital Imaging and Communications in Medicine dataset. The analyzed area for determining VAT and SAT was defined as the area from the top of the diaphragm to the navel level in the CT axial view. We also calculated the VAT to SAT (V/S) ratio to see how VAT-dominant or SAT-dominant body composition affected the cytokine/chemokine expressions and the response to the treatment. These imaging analyses were performed using MATLAB software (Mathworks, Natick, MA) and an in-house imaging analysis program, Medical Executable for the Efficient and Robust Quantification of Adipose Tissue (MEERQAT), from MD Anderson's Department of Radiation Oncology. MEERQAT can distinguish VAT from SAT by providing the volume of each component within a pre-specified area of interest. MEERQAT's detailed computing process has been described by Parikh *et al.* 17.

### Statistical analysis

The sample size of 30 was prospectively generated based on the estimated effect size and power calculation. With 30 patients, the power to test the null hypothesis of no correlation between variables ( 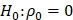 ) against moderate correlation between variables (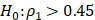 ) is 71%. Frequencies and percentages are reported for categorical variables. The Wilcoxon rank sum test and the Kruskal-Wallis test were used to compare the distributions of the continuous variables between the study groups. The Shapiro-Wilk test was performed to evaluate normality in continuous data. The Spearman correlation coefficient (SCC) was used to estimate correlations, which was based on the rank of the data and was robust to outliers and the normal distribution assumption. All tests were 2-sided. *P* values less than 0.05 were considered statistically significant without adjustment for multiple analyses. All analyses were conducted using SAS software, version 9.4.

## Results

### Patient characteristics

Patient characteristics are summarized in **Table 1**. The median BMI was 30.4, and 14 (50%) of the patients were categorized as obese according to the World Health Organization's BMI classifications. Seventeen (61%) of all patients were diagnosed with clinical stage III breast cancer, and 6 (21%) were diagnosed with clinical stage IV breast cancer. Eleven (39%) patients had the estrogen receptor (ER)+/human epidermal growth factor receptor 2 (HER2) negative subtype, 7 (25%) had the triple-receptor negative breast cancer (TNBC) subtype, 6 (21%) had the HER2 subtype, and 4 (14%) had the ER+/HER2+ subtype. Nine of these patients (32%) had inflammatory breast cancer.

### CAML profiles and subtypes

The average number of CAMLs per 7.5 ml of blood (*P* = 0.91), the average size of CAMLs (*P* = 0.44), and the maximum CAML size (*P* = 0.47) were not significantly different among the 4 molecular subtypes (ER+/HER2-, ER+/HER2+, triple-negative breast cancer, and HER2; the *P* values were based on the Kruskal-Wallis test).

### BMI and CAML profile correlations

The correlations between BMIs and CAML profiles are shown in **Table 2**. BMI was significantly positively correlated with the maximum size of CAMLs (SCC = 0.43; *P* = 0.035). When patients were divided into normal (BMI ≤ 25) and overweight/obese (BMI > 25) groups, the overweight/obese patients with breast cancer had a significantly higher average CAML size (*P* = 0.028) and maximum CAML size (*P* = 0.038) than did the patients with normal BMI levels **(Table 3)**. Furthermore, to estimate the odds ratio (OR) of the association between BMI and the maximum CAML size, the continuous maximum CAML size data was dichotomized into two levels (high vs low) using the median of 58 as the cutoff. Overweight and obese patients exhibited a 9.61times higher likelihood of having a high-level maximum CAML size (≥58) compared to patients with an underweight or normal BMI (95% CI of OR =1.54 - Infinity; *P* = 0.019, exact logistic regression analysis).

### Body composition and CAML profile correlations

The correlations between the CAML profiles and the body composition measurements ‒ the VAT amount, the SAT amount, and the V/S ratio ‒ were not statistically significant (all *P* >0.05), although the SAT amount trended towards correlation with the maximum CAML size (SCC = 0.364; *P* = 0.088; **Table 4**).

### Proinflammatory cytokines/chemokines and obesity correlations

The correlation between BMI and the expression of cytokines is shown in **Table 5**. M-CSF expression was significantly positively correlated with BMI; specifically, patients with a higher BMI level had increased M-CSF levels (SCC = 0.50; *P* = 0.0074). There was a marginal positive correlation between the MIP-1α level and BMI (SCC = 0.35; *P* = 0.068).

The other cytokines were not significantly associated with BMI. However, there was a trend that obese patients with breast cancer were more likely than nonobese patients to have higher levels of TNF-α, MIP-1α, and M-CSF (*P* <0.10). The body composition analysis showed that the M-CSF and SAT levels were significantly correlated (SCC = 0.48 *P* = 0.0104). No other significant correlations were found between the body composition parameters and the cytokines** (Supplemental Table 1)**.

### Proinflammatory cytokines/chemokines and CAML correlations

The correlations between CAML sizes and numbers and cytokines were not statistically significant (all *P* >0.05**; Supplemental Table 2**). However, the correlation between M-CSF and the average size of CAMLs was marginally significant (SCC = 0.38; *P* = 0.06). In the chemokine analysis, MIP-1α was significantly correlated with the average CXCR4 expression on CAMLs (SCC = 0.593; *P* = 0.003) and marginally correlated with the maximum CXCR4 expression on CAMLs (SCC = 0.353; *P* = 0.098). No significant correlations were found between the other cytokines and CXCR4 expression (**Supplemental Table 3**).

## Discussion

This is the first study to investigate the role of CAMLs on systemic inflammation and macrophage-related cytokines in obese patients with advanced breast cancer. We demonstrated a statistically significant correlation between BMI and the maximum size of CAMLs, BMI and M-CSF expression, and SAT-dominant body composition and M-CSF. We also found marginal correlations between BMI and MIP-1α, M-CSF and the average size of CAMLs, and maximum CXCR4 expression on CAMLs and MIP-1α. Although these marginal correlations were not statistically significant, they provided potential keys to understanding the biology of CAMLs in obese patient with advanced breast cancer.

The present study showed a significant positive correlation between BMI and the maximum CAML size. Also, a marginal correlation was observed between BMI and average CAML size. Furthermore, the detailed body composition analysis demonstrated a trend toward positive correlation between the SAT amount and the maximum CAML size. These results suggest that the size of CAMLs was associated with obesity or SAT-dominant body composition type. The SAT is the largest fat depot in the human body and is enriched by white adipose tissue that promotes chronic inflammation in obese environments. In an obese environment, the adipocytes can expand to up to 20 times larger than their normal size which increases their oxygen demand and leads to local hypoxia. Adipocytes in the hypoxic condition secrete hypoxia-inducible factor 1-alpha, which directly upregulates the production of proinflammatory cytokines such as TNF-α, IL-6, and MCP-1 18. These cytokines attract various immune cells, including macrophages, to the adipose tissue, and the macrophages also help establish the proinflammatory TME 19. Intriguigly, a previous report indicated that the origin of CAMLs is the macrophages in the TME 10, which help establish obesity-induced proinflammation states. Our study result implied the link between macrophages in TME and CAMLs through the obesity-induced imflammation.

Although the present study indicated that the size of the CAMLs was associated with SAT-dominant body composition or obesity, it is unclear why there was no significant difference in the number of CAMLs in overweight and obese vs. normal-weight patients. A previous study including various types of solid tumor showed that the average CAML size increased as the clinical stage increased 20. A similar trend of the number of CAMLs increasing as the clinical stage increased was also seen in the study, although the CAML number was not significantly different for patients with stage I vs. stage II cancer 20. This result may indicate that the size of CAMLs increases before the number of CAMLs increases as cancer progresses. Indeed, a previous study demonstrated that CAMLs ingest necrotic tumor debris in patients with pancreatic or prostate cancer by detecting the expression of tumor-specific antigens inside the CAMLs 10. Thus, increased CAML size could be due to their activated phagocytic activity caused by the pro-inflammatory environment.

The macrophage-related cytokines M-CSF and MIP-1α and the pro-inflammatory cytokine TNF-α had significant or marginal positive correlations to BMI, and M-CSF had a marginal correlation to the SAT amount and average CAML size. M-CSF is a primary growth factor that regulates tumor growth, proliferation, and cell differentiation in hematopoietic lineages, including macrophages 21. It is secreted from tumor and stromal cells, such as CAMLs, and from white adipose tissue, which is a major source of M-CSF production 22. M-CSF has a direct effect on the activation of the PI3K and MAPK signaling pathways via the colony-stimulating factor-1 receptor. Furthermore, M-CSF recruits macrophages into the TME which promotes angiogenesis and tumor growth 21. In this study, we also observed a trend toward high expression of MIP-1α, a chemotactic chemokine produced by macrophages, in obese patients with breast cancer; this is a sign of macrophage activation. MIP-1α has various biological functions, including recruiting inflammatory cells, aiding in wound healing, inhibiting stem cells, and maintaining the effector immune response 23. Although the present study did not investigate the interaction between M-CSF and MIP-1α in the TME, coexpression of those markers in obese patients with breast cancer indicated the activation and active trafficking of TAMs. Another essential biological finding in this study is the correlation between the CXCR4 expression on CAMLs and MIP-1α expression. CXCR4 is a receptor of CXCL12 (also known as stromal cell-derived factor 1), which is often expressed in breast cancer, and high CXCR4 expression is an indicator of poor survival prognosis 24, 25. Importantly, the activation of the CXCR4/CXCL12 signaling pathway differentiates monocytes into proangiogenic, immunosuppressive macrophages in the TME 16. No previous study has evaluated CXCR4 expression in circulating cells; accordingly, future studies should clarify whether the expression of CXCR4 on CAMLs represents the active differentiation of monocytes into macrophages in the TME.

In summary, the present study results generates a new hypothesis that, in the SAT-dominant body composition or obesity environment, various cells, including white adipocytes, tumor cells, and stromal cells, secrete M-CSF and MIP-1α, leading to the recruitment of macrophages to the TME. The recruited macrophages then help establish chronic inflammation and phagocytose tumor debris and dead adipocytes in the TME. These macrophages may return to the peripheral circulation as CAMLs expressing CXCR4.

The present study has several limitations. First, the small sample size of 28 prevents definitive conclusions. Also, p-values and level of significant were not adjusted for multiple analysis, increasing the risk of false positive results. The original sample size (30) was generated based on the estimated effect size and power calculation. However, the present study could not show a large effect size as originally estimated, and the obtained result is underpowered. Second, as mentioned above, the present study included patients with advanced and de novo stage IV disease, including Inflammatory Breast Cancer, which might have created inconsistencies in the study results. Future studies should involve a cohort with a standardized clinical background (i.e., clinical stage and receptor subtype) or a patient cohort large enough to be stratified by clinical background. Last, the present study only investigated the CAMLs and cytokine/chemokines in the systemic circulation, not in the TME. Therefore, the biological role of CAMLs in TME is still unknown. Further investigation of the TME including spatial analysis is needed to confirm the origin of CAMLs, their biological behavior, and their interaction with other cells such as immunocytes, adipocytes, and fibroblasts. The retrospective review of archival primary tumor tissues could be useful for this purpose.

## Conclusions

The present study discovered that the size of CAMLs may be associated with SAT-dominant obesity. Furthermore, increased macrophage-related markers and proinflammatory markers were observed in obese patients with breast cancer. These findings may imply an association between CAMLs and activated macrophages in the TME and may contribute to a better understanding of the mechanism of obesity-induced breast cancer progression and, potentially, to biomarker development.

## Supplementary Material

Supplementary tables.

## Figures and Tables

**Table 1:** Patient characteristics

Variable	Number of patients(N = 28)	%
Median age, years (range)	48.5 (25-75)	
Race/ethnicity		
White	13	46
African American	5	18
Hispanic	9	32
Asian	1	4
Reproductive status		
Premenopausal	11	39
Menopausal	13	47
Unknown	4	14
Median BMI, kg/m^2^ (range)	30.4 (20.7-60.5)	
WHO BMI classification		
Underweight	0	0
Normal	6	21
Overweight	8	29
Obese	14	50
TNM clinical stage		
I	0	0
II	5	18
III	17	61
IV	6	22
Breast cancer subtype		
ER+/HER2-	11	39
ER+/HER2+	4	14
TNBC	7	25
HER2	6	22
IBC		
Yes	9	32
No	19	68

Abbreviations: BMI, body mass index; ER, estrogen receptor; HER2, human epidermal growth factor receptor 2; IBC, inflammatory breast cancer; TNBC, triple-negative breast cancer; WHO, World Health Organization.

**Table 2:** BMI and CAML profile correlations

		CAML profile		
		Number	Average size	Maximum size
**BMI**	SCC	0.17	0.39	0.43
	*P*	0.38	0.059	0.035
	Number of observations	28	24	24

*P was based on the Spearman correlation test. Abbreviations: BMI, body mass index; CAML, cancer-associated macrophage-like cell; SCC, Spearman correlation coefficient.

**Table 3:** Association between WHO BMI classifications and CAML profiles

CAML profile	BMI	Number of patients (N = 28)	Mean ± standard deviation	*P*
**Number of CAMLs**	Normal	6	1.3 ± 0.8	0.567
	Overweight/obese	22	2.9 ± 3.3	
**Average CAML size**	Normal	5	34.1 ± 7.6	00.028
	Overweight/obese	19	59.2 ± 21.1	
**Maximum CAML size**	Normal	5	35.4 ± 8.3	0.038
	Overweight/obese	19	77.3 ± 39.0	

Abbreviations: BMI, body mass index; CAML, cancer-associated macrophage-like cell; WHO, World Health Organization.

**Table 4:** Correlation between CAML profiles and body composition parameters

		CAML profiles		
		Number	Average size	Maximum size
**VAT amount**	SCC	0.09	0.12	0.30
	P*	0.648	0.606	0.159
	Number of observations	27	23	23
**SAT amount**	SCC	0.05	0.29	0.36
	*P*	0.817	0.18	0.088
	Number of observations	27	23	23
**V/S ratio**	SCC	0.09	-0.20	0.02
	*P*	0.669	0.349	0.918
	Number of observations	27	23	23

*P was based on the Spearman correlation test. Abbreviations: CAML, cancer-associated macrophage-like cell; SAT, subcutaneous adipose tissue; SCC, Spearman correlation coefficient; VAT, visceral adipose tissue; V/S, visceral adipose tissue to subcutaneous adipose tissue.

**Table 5:** Correlation between BMI and the expression of cytokines

Variables (N = 28)								
Variable	BMI	CXCL12	IL-1b	IL-6	TNF-α	MIP-1α	MIP-1β	M-CSF
**BMI** SCC	1.00	0.06	0.23	0.12	0.17	0.35	-0.03	0.50
P*	N/A	0.759	0.230	0.543	0.388	0.068	0.886	.007
**CXCL12** SCC		1.00	0.11	-0.15	0.51	0.33	0.16	0.24
P*		N/A	0.569	0.438	0.006	0.087	0.416	0.219
**IL-1b** SCC			1.00	-0.18	0.23	0.13	0.16	0.04
P*			N/A	0.371	0.240	0.498	0.424	0.832
**IL-6** SCC				1.00	0.00	0.20	-0.14	0.40
P*				N/A	0.986	0.320	0.475	0.034
**TNF-α** SCC					1.00	0.44	0.57	0.44
P*					N/A	0.021	0.001	0.018
**MIP-1α** SCC						1.00	0.25	0.52
P*						N/A	0.190	0.005
**MIP-1β** SCC							1.00	-0.09
P*							N/A	0.660
**M-CSF** SCC								1.00
P*								N/A

*P was based on the Spearman correlation test. Abbreviations: BMI, body mass index: CXCL12, C-X-C motif chemokine ligand 12; IL, interleukin; M-CSF, macrophage-colony stimulating factor; MIP, macrophage inflammatory protein; N/A, not applicable; SCC, Spearman correlation coefficient; TNF-α, tissue necrosis factor-alpha.

## Abbreviations

CAMLs: cancer-associated macrophage-like cells
CXCR4: C-X-C chemokine receptor type 4
BMI: body mass index
M-CSF: macrophage-colony stimulating factor
SAT: subcutaneous adipose tissue
TME: tumor microenvironment
cluster of differentiation: CD
tumor-associated macrophages: TAMs
NAC: neoadjuvant chemotherapy
CT: computed tomography
EDTA: ethylenediaminetetraacetic acid
IL: interleukin
TNF-α: tissue necrosis factor-alpha
MIP: macrophage inflammatory protein
DAPI: 4′,6-diamidino-2-phenylindole
WBCs: white blood cells
CXCL12: C-X-C motif chemokine ligand 12
VAT: visceral adipose tissue
V/S: visceral adipose tissue to subcutaneous adipose tissue
MEERQAT: Medical Executable for the Efficient and Robust Quantification of Adipose Tissue
SCC: Spearman correlation coefficient
ER: estrogen receptor
HER2: human epidermal growth factor receptor 2
TNBC: triple-receptor negative breast cancer
SCC: Spearman correlation coefficient
WHO: World Health Organization
N/A: not applicable
