# Supplementary material for: Circulating cancer-associated macrophage-like cells and macrophage-related cytokines in obese patients with advanced breast cancer who undergo neoadjuvant chemotherapy
Source: J Cancer. 2024 Sep 16;15(18):5855–62. doi: 10.7150/jca.89453 (PMC11493001; doi:10.7150/jca.89453)
Supplement: Supplementary file 1 — Supplementary tables. [file jcav15p5855s1.pdf]

Supplemental Table 1. Correlation between body composition and cytokines

Variables			
**Cytokine**	**Amount of VAT**	**Amount of SAT**	**V/S ratio**
**CXCL12**			
SCC	0.11	0.20	-0.07
*P***	0.588	0.329	0.718
Number of observations	27	27	27
**IL-1b**			
SCC	0.17	0.24	−0.05
*P***	0.403	0.221	0.8122
Number of observations	27	27	27
**IL-6**			
SCC	−0.09	−0.13	−0.09
*P***	0.641	0.503	0.660
Number of observations	27	27	27
**TNF-α**			
SCC	0.10	0.15	−0.04
*P***	0.617	0.463	0.862
Number of observations	27	27	27
**MIP-1α**			
SCC	0.13	0.28	−0.02
*P***	0.527	0.159	0.921
Number of observations	27	27	27
**MIP-1β**			
SCC	−0.23	−0.13	−0.25
*P***	0.253	0.524	0.214
Number of observations	27	27	27
**M-CSF**			
SCC	0.25	0.48	−0.13
*P***	0.208	0.010	0.522
Number of observations	27	27	27

**P* was based on the Spearman correlation test.

Abbreviations: CXCL12, C-X-C motif chemokine ligand 12; IL, interleukin; M-CSF, macrophage-colony stimulating factor; MIP, macrophage inflammatory protein; SAT, subcutaneous adipose tissue; TNF-α, tissue necrosis factor-alpha; VAT, visceral adipose tissue; V/S, visceral adipose tissue to subcutaneous adipose tissue.

Supplemental Table 2. Correlation between CAML profiles and the expression of cytokines

Cytokine		Number of CAMLs	Average size of CAMLs	Maximum size of CAMLs
**CXCL12**	SCC	−0.22	−0.15	−0.16
	P	0.25	0.49	0.45
	Number of observations	28	24	24
**IL-1b**	SCC	−0.11	0.05	0.07
	P	0.57	0.80	0.75
	Number of observations	28	24	24
**IL-6**	SCC	0.10	0.28	0.32
	P	0.60	0.19	0.13
	Number of observations	28	24	24
**TNF-α**	SCC	0.11	−0.01	0.06
	P	0.58	0.97	0.79
	Number of observations	28	24	24
**MIP-1α**	SCC	0.01	−0.06	0.00
	P	0.95	0.77	0.99
	Number of observations	28	24	24
**MIP-1β**	SCC	0.13	0.20	0.22
	P	0.50	0.34	0.29
	Number of observations	28	24	24
**M-CSF**	SCC	0.02	0.38	0.32
	P	0.92	0.06	0.12
	Number of observations	28	24	24

Abbreviations: CXCL12, C-X-C motif chemokine ligand 12; IL, interleukin; M-CSF, macrophage-colony stimulating factor; MIP, macrophage inflammatory protein; SCC, Spearman correlation coefficient; TNF-α, tissue necrosis factor-alpha.

Supplemental Table 3. Correlation between CXCR4 expression and cytokines

Variables		
**Cytokine**	**Average CXCR4 expression on CAMLs**	**Maximum CXCR4 expression on CAMLs**
**CXCL12**		
SCC	−0.03756	−0.19427
P	0.8649	0.3744
Number of observations	23	23
**IL-1b**		
SCC	0.32756	0.31774
P	0.1271	0.1396
Number of observations	23	23
**IL-6**		
SCC	−0.03882	0.09161
P	0.8604	0.6776
Number of observations	23	23
**TNF-α**		
SCC	0.16753	0.03559
P	0.4448	0.8719
Number of observations	23	23
**MIP-1α**		
SCC	0.59333	0.35336
P	0.0028	0.0981
Number of observations	23	23
**MIP-1β**		
SCC	−0.02174	−0.06425
P	0.9216	0.7709
Number of observations	23	23
**M-CSF**		
SCC	0.07609	−0.02273
P	0.7301	0.918
Number of observations	23	23

Abbreviations: CAML, cancer-associated macrophage-like cell; CXCL12, C-X-C motif chemokine ligand 12; CXCR4, C-X-C chemokine receptor type 4IL, interleukin; M-CSF, macrophage-colony stimulating factor; MIP, macrophage inflammatory protein; SCC, Spearman correlation coefficient; TNF-α, tissue necrosis factor-alpha.
